# Operationalization of assent for research participation in pre-adolescent children: a scoping review

**DOI:** 10.1186/s12910-022-00844-2

**Published:** 2022-11-03

**Authors:** Florence Cayouette, Katie O’Hearn, Shira Gertsman, Kusum Menon

**Affiliations:** 1 Division of Critical Care, Department of Pediatrics, Children’s Hospital of Eastern Ontario, University of Ottawa, Ottawa, ON Canada; 2 CHEO Research Institute, Children’s Hospital of Eastern Ontario, Ottawa, ON Canada; 3 Present Address: Faculty of Medicine, McMaster University, Hamilton, ON Canada

**Keywords:** Assent, Children, Capacity

## Abstract

**Background:**

Seeking assent from children for participation in medical research is an ethical imperative of numerous institutions globally. However, none of these organizations provide specific guidance on the criteria or process to be used when obtaining assent. The primary objective of this scoping review was to determine the descriptions of assent discussed in the literature and the reported criteria used for seeking assent for research participation in pre-adolescent children.

**Methods:**

Medline and Embase databases were searched until November 2020 using the term “assent” in the title or abstract. Inclusion criteria were (1) studies enrolling children which specifically described operationalization of the assent process and (2) studies of the assent process which provided a description of assent. Data collected included participant information, patient criteria for seeking assent, guidelines referenced, description of assent reported, how assent was obtained and assent information presented, and reported assent rate. For qualitative articles focusing on the assent process, important themes were identified.

**Results:**

A total of 116 articles were included of which 79 (68.9%) operationalized assent and 57 studies (%) described the assent process. The most commonly reported criterion used to determine the ability of a child to assent was age (35.4%, 28/79). The reported minimal age for obtaining pediatric assent varied considerably across and within jurisdictions (5–13 years; median 7.5 years, IQR 7.0, 9.75). Cognitive ability was reported as a criterion for obtaining assent in 5.1% (4/79) of studies. Assent rates were only reported in 17.7% (14/79) of citations and ranged from 32.0 to 100%. Analysis of the 57 studies describing the assent process identified several themes, including age thresholds, assessment of capacity, variable knowledge of pediatric assent and parental roles.

**Conclusion:**

We found significant variation in criteria used for assessment of patient capacity, delivery of information used to obtain assent and documentation of the assent process. While we acknowledge that individual children, settings and jurisdictions may require different approaches to obtaining assent, there should be agreement on important principles to be followed with resulting common guidance on assessing capacity, delivering information and documentation of the assent process for publication.

**Supplementary Information:**

The online version contains supplementary material available at 10.1186/s12910-022-00844-2.

## Introduction

Respecting the rights of children is important when conducting clinical research. As per the Declaration of Helsinki [1], “When a potential research subject who is deemed incapable of giving informed consent is able to give assent to decisions about participation in research, the physician must seek that assent in addition to the consent of the legally authorized representative”. In addition, Article 12 of the United Nations Convention on the Rights of the Child states, “Parties shall assure to the child who is capable of forming his or her own views the right to express those views freely in all matters affecting the child” [2]. In addition, several pediatric societies have stated that children must be given the opportunity to provide assent when able to [3, 4]. However, operationalizing these mandates is challenging given the age and developmental constraints of conducting research in pediatric populations.

Health Canada and the Public Health Agency of Canada state that “A child under 16 years of age should provide his/her assent and may refuse to participate even if the parent has provided their consent” but does not specify a developmental age or process for obtaining assent [5]. Similarly, the United States Department of Health and Human Services states that research in children must involve the “use of parental permission and child assent” [6] but again does not recommend a specific age or provide further guidance. The House of Lords in England held that “a child under 16 had the legal competence to consent to medical examination and treatment if they had sufficient maturity and intelligence to understand the nature and implications of that treatment” [7]. However, this law specifically pertains to medical treatment and does not address the issue of consent or assent for research in minors.

In addition, obtaining assent in children presents challenges that differ across the age spectrum. Although jurisdictions vary significantly in the legal ability of adolescents to provide consent for research, many experts acknowledge the ability of adolescents to understand medical information presented to them [8]. The implicit assumption that adolescents can understand medical information is further supported by the mandate of most research ethics boards requiring consent forms to be written at a Grade 8 level [9]. Therefore, while adolescent decision-making poses unique and important challenges such as emerging autonomy, impulsivity [10] and potential emancipation, these challenges differ significantly from those encountered when approaching younger children for assent and are thus best addressed separately.

In view of ethical mandates for obtaining assent from minors for participation in medical research and the paucity of specific guidelines for doing so, the objective of this scoping review was to determine (1) descriptions of the assent process discussed in the literature, (2) reported criteria used for seeking assent, (3) methods in which information for obtaining assent is provided and (4) documentation of assent procedures in pre-adolescent children.

## Methods

### Study selection and search strategy

The Medline database was searched from inception (1966) to November 2020 and Embase from 1998 to November 2020 using the word “assent” in the title or abstract. A random sample of 100 articles with the word “assent” in the full-text but not in the title or abstract demonstrated only one article containing a further description of the assent process within the body of the article. This provided justification for our pragmatic approach of only including studies with “assent” in the title or abstract. The date limit of 1998 was chosen for the Embase search as the Canadian Tri-Council Policy Statement [11] was implemented in 1998 and resulted in significant changes to ethical guidelines for research. This was reflected in the Medline search which demonstrated less than 8% of articles retrieved were from prior to 1998.

### Selection criteria

Inclusion criteria were (1) studies focusing on pediatric assent if they provided or generated a description of the assent process and (2) studies enrolling children which specifically described operationalization of the assent process. Randomized controlled trials (RCTs), cohort studies, case-control studies, cross-sectional surveys, case reports, patient registries, focus groups and study protocols in English or French were considered. Neonatal literature, narrative reviews and editorials were excluded. In addition, articles focusing solely on adolescents or youth (as defined by the authors) or participants ≥ 12 years of age were excluded. Adolescent populations were excluded as previously outlined as ethical issues in this group differ significantly from those of younger children [12] and were therefore better served by a separate review.

### Data extraction and analysis

Study demographics were collected for all citations. For clinical studies enrolling children, data collected included participant information, guidelines referenced, description of assent reported, how assent was obtained and assent information presented, age criteria and other exclusion criteria, and reported assent rate. The following information was collected and themes identified for citations generating a description of the assent process: specific assent focus, understanding of the assent process, the way assent is carried out in practice, and improvements or innovation tools suggested. Title, abstract and full text review were performed by two independent reviewers using InsightScope [13]. Data was extracted by two independent reviewers using REDcap (research electronic data capture) tools [14]. Disagreements were resolved by consensus or if consensus could not be achieved, by a third reviewer.

Study demographics were collected for all citations. The following information was collected and themes identified for citations generating a description of assent: specific assent focus, understanding of the assent process and the way assent is carried out in practice. For clinical studies enrolling children, data collected included participant information, guidelines referenced, description of assent reported, how assent was obtained and assent information presented, age criteria and other exclusion criteria, and reported assent rate. Since our scoping review did not involve the determination of a treatment effect or comparison between groups, a meta-analysis was not conducted. A descriptive analysis of demographic data, participant information, methods for obtaining assent and providing information was conducted. Median values along with interquartile ranges for the minimal age required for assent and reported assent rates were calculated.

## Results

**Fig. 1:**
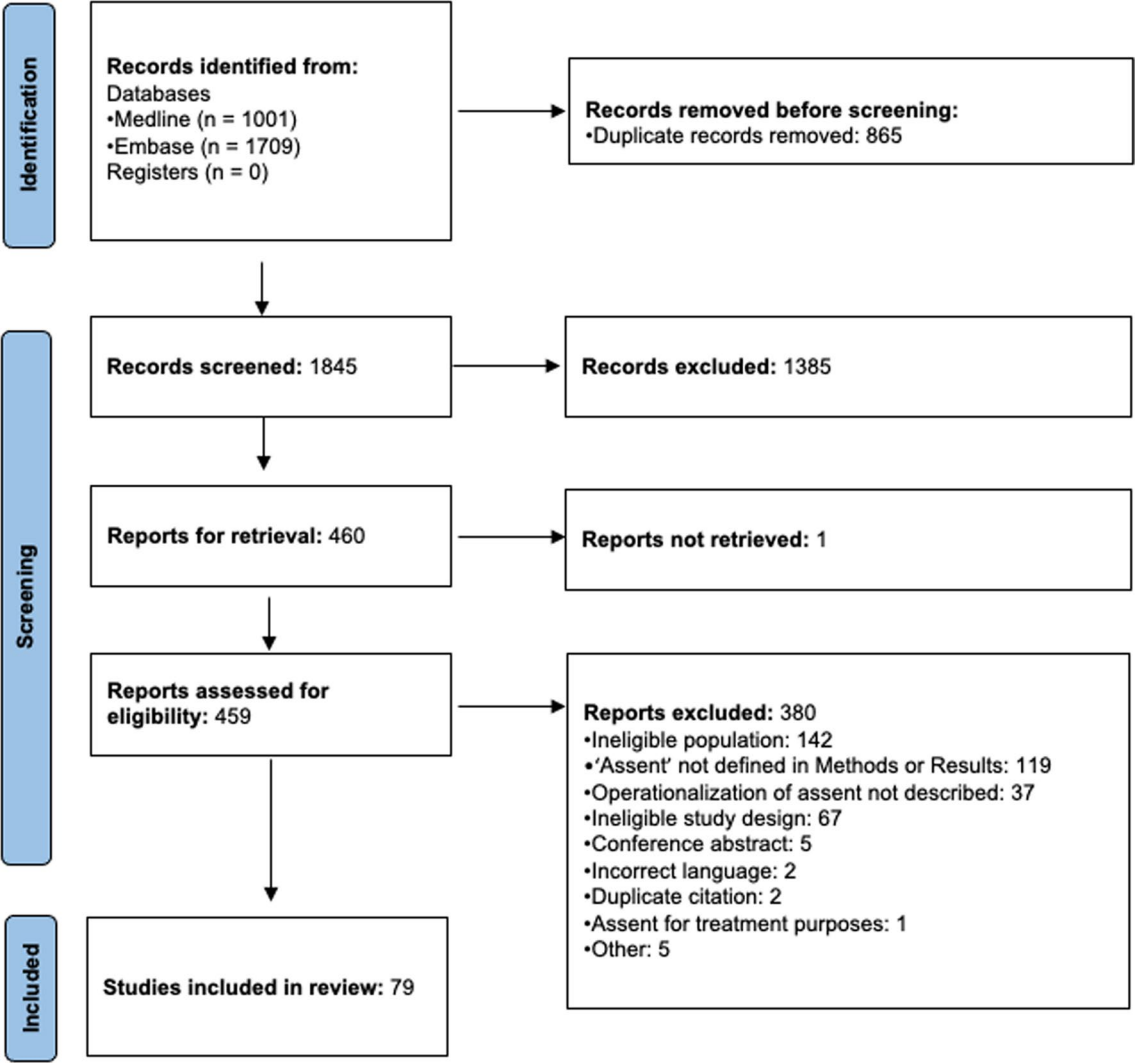
PRISMA flow diagram

A total of 2710 records were identified through our search strategy. After duplicates were removed, 1845 records were screened and 1385 records excluded after title and abstract review. Full-text review of the retrievable 459 citations yielded 79 eligible studies. The PRISMA flow diagram is shown in Fig. 1. The references for the 45 out of 79 included citations that are not listed in the main manuscript are attached in Additional file 1.

### Characteristics of included studies

Characteristics of the included citations are detailed in Table 1. Of note, no study which reported on operationalizing assent and only two studies describing the assent process were published before 1990.

**Table 1:** Characteristics of included citations

	Studies operationalizing assent (n = 79) Number (%)	Studies defining assent (n = 57) Number (%)
Year of publication		
1990–2000	2 (2.5)	2 (3.5)
2000–2010	15 (19.0)	18 (31.6)
2010–2020	62 (78.5)	35 (61.4)
Location of main study site^a^		
North America	43 (54.4)	32 (56.1)
Europe	14(17.7)	18 (31.6)
Africa	12 (15.2)	4 (7.0)
Central and South America	5 (6.3)	2 (3.5)
East Asia	1 (1.3)	2 (3.5)
Rest of Asia	6 (7.6)	0 (0)
Australia/New Zealand	3 (3.8)	2 (3.5)
Middle East	0 (0)	1 (1.8)
Number of centres		
Single-center	54 (68.4)	31 (54.4)
Multi-center	20 (25.3)	12 (21.1)
International sites: yes	6 (7.6)	5 (8.8)
International sites: no	14 (17.7)	7 (12.3)
Not reported	2 (2.5)	6 (10.5)
Not applicable	3 (3.8)	8 (14.0)
Study design^b^		
Systematic/scoping review	0 (0)	8 (14.0)
Randomized controlled trial	13 (16.5)	2 (3.5)
Quasi-randomized studies	4 (5.1)	1 (1.8)
Cohort study	38 (48.1)	15 (26.3)
Case-control study	1 (1.3)	0 (0)
Survey/interview/focus group	15 (19.0)	30 (52.6)
Patient registry	1 (1.3)	0 (0)
Study protocol	8 (10.1)	0 (0)
Other^c^	4 (5.1)	6 (10.5)

^a^Articles were classified by location of the main study site, using the Standard Country or Area Codes for Statistical Use from the Statistics Division of the United Nations. Some studies could have more than one main site

^b^Some studies used more than one study design

^c^These included: validation of diagnostic test, mixed methods study, feasibility study and methodologic papers on the need for a randomized controlled trial

#### Studies focusing on the description of assent

The main characteristics of the 57 studies generating or providing a description of assent are summarized in Table 2.

**Table 2:** Characteristics of studies defining assent (n = 57)

	Number (%)
*Reason for obtaining assent*	
Research purposes	50 (87.7)
Clinical purposes	6 (10.5)
Organ donation^a^	2 (3.5)
*Context of the study on assent*	
For research/treatment in general (may include guidelines)	21 (36.8)
In a specific real study/treatment performed on children	16 (28.1)
Across multiple real studies/treatments	12 (21.1)
In a specific hypothetical study/treatment	3 (5.3)
Other^b^	5 (8.8)
*Specific assent focus*	
Opinions/perceptions about assent	30 (52.6)
Practicalities of the assent process	15 (26.3)
Guidelines	13 (22.8)
Improvement/innovation tools	12 (21.1)
Recall/understanding of assent information	10 (17.5)
Other^c^	3 (5.3)

^a^Related to children’s opinions about organ donation and to minors living organ donation

^b^Other contexts included HIV/AIDS research, multiple hypothetical studies, genetic testing research and pediatric biobanking

^c^Other assent focuses included decision-making processes, ethical and procedural issues surrounding pediatric assent and sociodemographic characteristics of children who assent or dissent

The studies were classified based on their main assent focus, which we divided as guidelines, opinions and perceptions, recall and understanding of the assent process, practicalities of the process and improvement or innovation tools.

In the 30 studies focusing on opinions and perceptions of assent, the main populations studied were children (60%), parents (23.3%) [23, 26–31], researchers (23.3%) [15, 17, 29, 30, 32–34] and healthcare professionals (16.7%) [15, 22, 23, 30, 31]. The main themes identified in these studies were: children’s desire to be involved and their empowerment [24–26, 32, 35–39], need for cultural considerations [12, 17, 18, 26, 28, 30, 31, 40], specific context considerations and opinion variation [21–23, 29, 33, 34, 41] as well as need for age and developmental considerations [13, 27, 42–45].

Fifteen studies focused on pediatric assent practicalities and identified complexity and variability of assent practices [23, 46], variability of clinicians and researchers’ knowledge about assent [22, 33] as well as cultural and context-specific assent considerations [17, 18, 29, 30, 34, 47] as important themes.

Thirteen studies had a main focus on pediatric assent guidelines. Of these, 53.8% were institutionally based [12, 19, 48–51], 30.8% were study-specific [19, 29, 46, 51] and 23.1% were national or international guidelines [12, 14, 16]. The majority (76.9%) specified a minimal age, ranging from 4 to 12 years, above which assent should be obtained [14, 16, 19, 46, 48–51]. The majority of the studies referred to North American (61.5%) [12, 19, 46, 48–51] and European guidelines (38.5%)[12, 14, 16, 29, 46]. In 84.6% of the studies, the guidelines did not specify in what manner assent should be obtained (for example written or verbal) and 76.9% did not specify how information should be presented. Only 30.8% of these studies specified what information was provided to research participants. This information included, for example, common risks and benefits, potential serious side effects, description of research procedures and informing children that they can refuse to participate [12, 29]. One study provided information by age category to ensure age and developmental appropriateness of the language used.

Ten studies explored pediatric assent recall and/or understanding. The populations studied included children (100%) [21, 35, 39, 44, 45], parents (10%) and healthcare professionals (10%). One study concluded that “children less than 9 years old cannot provide assent in a meaningful way” [44], whereas another study concluded that children less than 11 years old have a limited understanding of research information. The need to develop tools to assess children’s understanding was emphasized in a study concluding that “most children have limited understanding of research” [45].

### Studies enrolling children

Research Ethics Board (REB) approval was reported in 93.7% of included studies (74/79).

**Table 3:** Characteristics of populations in studies enrolling children (n = 79)

REB approval reported	Number (%)
Yes	74 (93.6)
Not specified/unclear	5 (6.4)
* Study setting*^a^	
Out-patient	34 (43.0)
Hospital	20 (25.3)
School	18 (22.8)
Community	8 (10.1)
Not reported	2 (2.5)
Other^b^	14 (17.7)
* Primary language spoken *	
English	22 (27.7)
French	3 (3.8)
Spanish	4 (5.1)
Other	8 (10.1)
Not clearly stated	43 (54.4)
* Study patient population *	
Healthy children	22 (27.7)
Outpatient clinics	16 (20.3)
Hematology-oncology	7 (8.9)
Surgical	7 (8.9)
HIV infections	5 (6.3)
Dental	4 (5.1)
Hospital ward admissions	2 (2.5)
Emergency department	4 (5.1)
Other^c^	12 (15.2)

^a^Some studies were conducted in more than one setting

^b^Other settings included home, telephone, online, day-care, study clinic, public space and tribal villages

^c^Other study populations included cohorts of children with overweight/obesity, asthma, trauma, attention-deficit hyperactivity disorder, autism spectrum disorder, fetal alcohol spectrum disorder, traumatic brain injury and orthopedic injuries

The most common study settings were out-patient clinics (34/79, 43.0%), hospitals (20/79, 25.3%) and schools (18/79, 22.8%). The most common populations studied were healthy children (22/79, 27.7%) and those presenting to out-patient clinics (16/79, 20.3%). The majority of studies (43/79, 54.4%) did not specify the primary language spoken by study participants, but for those studies which did, English was the most commonly utilized language (22/30).

### Reported criteria for seeking assent in studies enrolling children

**Fig. 2:**
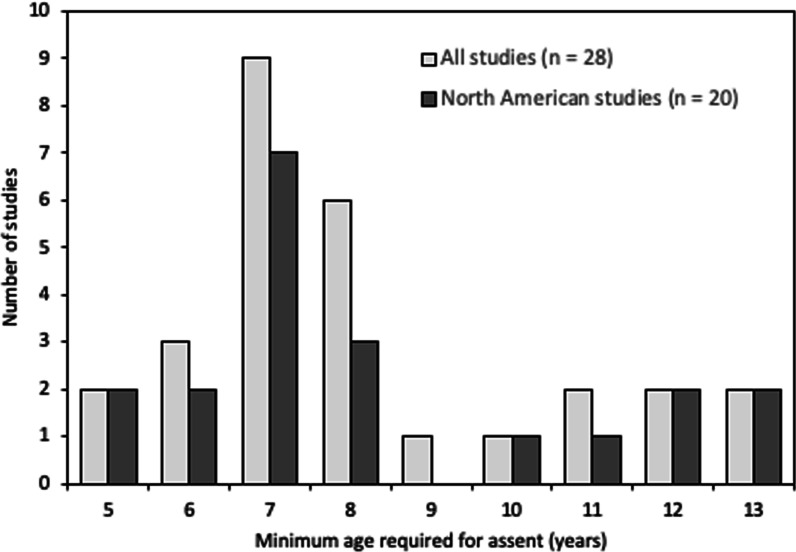
Minimum age specified for obtaining assent

**Fig. 3:**
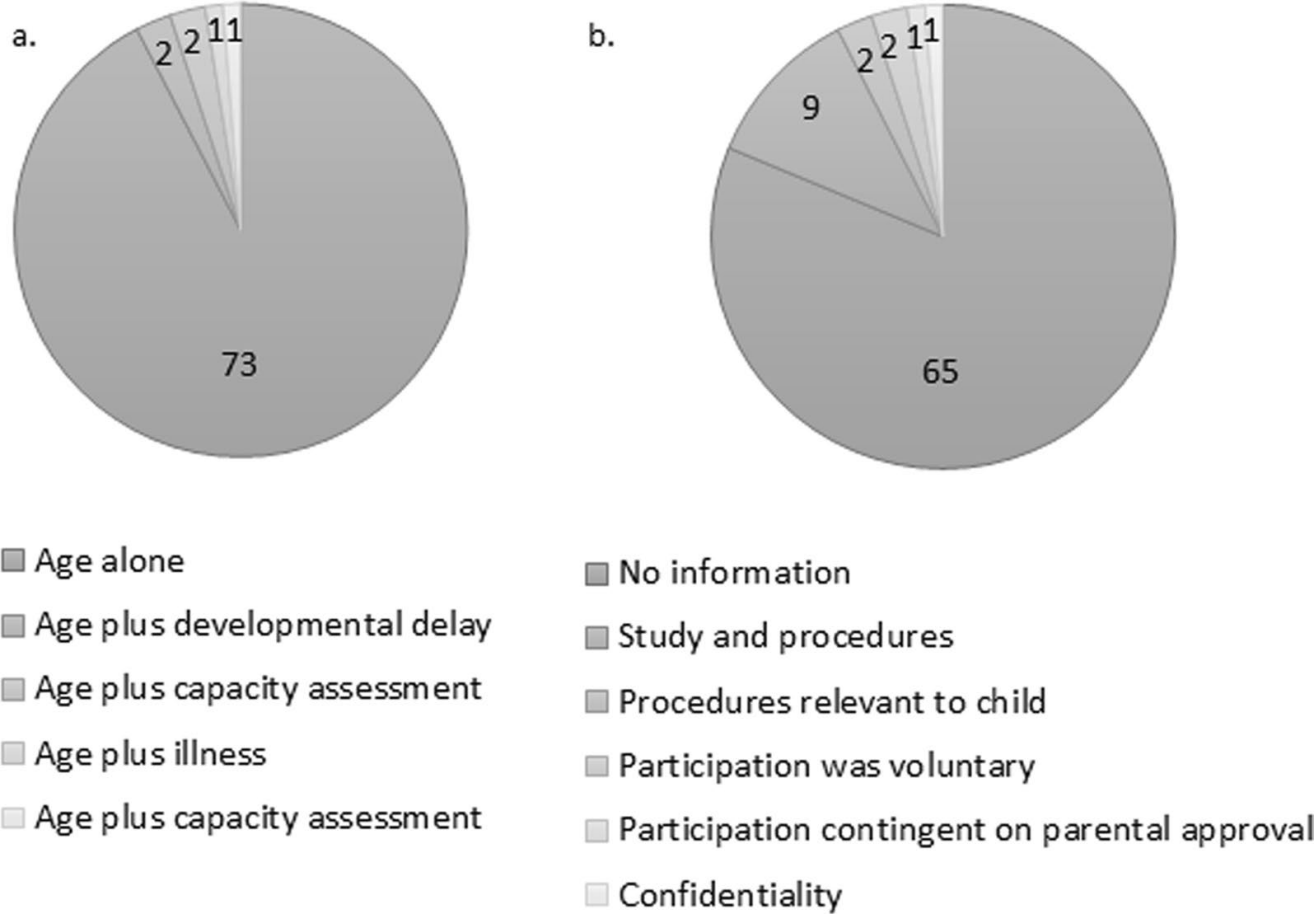
**a** Criteria used to determine ability to provide assent. **b** Information provided to child during the assent process

The most commonly reported criterion for seeking assent was a minimal age which was only specified in 35.4% (28/79) of studies and varied between 5 and 13 years (median 7.5 years, IQR 7.0–9.75; mode 7.0 years). Figure 2 shows the frequency of the reported minimum age required for obtaining assent across all studies (n = 28) and within the North American studies alone (n = 20). Seven studies specified criteria used for obtaining assent other than age (see Table 3) [15–21] and Fig. 3a. Of these, only four studies [15, 16, 18, 21] specifically reported using developmental disabilities or difficulty in understanding assent procedures as exclusion criteria for obtaining assent. Twelve studies (15.2%, 12/79) referenced ethical guidelines to inform their assent procedures [15, 22–32] and legal guidelines on obtaining assent were referenced in only one study which stated that the national children’s rights law was considered but did not provide specific details [22].

### Methods in which information for obtaining assent was provided

Reported assent processes were extremely variable (see Table 4). 41% of studies (33/79) did not specify how assent was obtained and 57.0% (45/79) did not specify in what format assent information was provided to children. When reported, the most common method of obtaining assent was written (31/53) and the most common method of providing information was verbal (20/34). Only 16.5% (13/79) of studies reported on the type of information to the patient for assent (see Fig. 3b).

**Table 4:** Assent processes used to enroll children into clinical studies (n = 79)

Method for obtaining assent^a^	
Not specified	33 (41.8)
Written	31 (39.2)
Verbal	18 (22.8)
Other^b^	4 (5.1)
Minimal age for assent specified	
Yes	28 (35.4)
Min (years)	5
Max (years)	13
No	51 (64.6)
Exclusion criteria other than age specified	
Yes^c^	6 (7.6)
No	73 (92.4)
Format assent information provided^d^	
Not specified	45 (57.0)
Verbal	20 (25.3)
Written in person	14 (17.7)
Written by mail	3 (3.8)
Written (not further specified)	4 (5.1)
Unclear	3 (3.8)
Comic book	1 (1.3)
Other^d^	3 (3.8)
Information provided to patient specified	
Yes	13 (16.5)
No	66 (83.5)
Assent rate reported or able to be calculated	
Yes	14 (17.7)
No	57 (72.2)
Not applicable (e.g. study protocol)	8 (10.1)
Assent rate (reported or calculated)	
Median (range)	89.0 (32.0-100.0)

^a^Some studies obtained assent in more than manner

^b^Other ways to obtain assent were via a form (but unclear if form used to get signature), nonverbal, use of an online form and formal acceptance

^c^Specific exclusion criteria were developmental disabilities or extreme difficulties in understanding simple instructions for assent procedure and baseline questionnaire, being an adolescent (not age-specified), being too unwell to participate, being in school grade less than 5 (not age-specified), failing comprehension assessment because of substance use and moderate or severe learning difficulties

^d^Some studies provided the information in more than one format

^e^Other formats used included cartoons/play, online written form and pictures that children were invited to color

### Documentation of assent procedures

The assent rate was reported in only 17.7% (14/79) of included studies and ranged from 32 to 100% (median 89.0%, IQR 78.5, 100) [15, 17, 22, 27, 31, 33–41]. The majority of studies did not comment on the concordance of the parental consent rate and child assent rate (12/14, 85.7%). Two of the 14 studies, however, reported that assent was not obtained and therefore the child not enrolled in 16.7% (14/84) [22] and 6.3% (2/32) [37] of potential participants respectively. Neither study discussed the implications of not obtaining assent for the results of their study.

## Discussion

Our review did not find consistently used principles or guidelines for obtaining assent from pre-adolescent children for participation in medical research. In addition, this scoping review found the criteria for obtaining assent and the approach to assent procedures in pre-adolescent populations varied significantly and were sparsely documented.

The most commonly reported criteria used when seeking assent was age. However, the reported minimal age required for obtaining pediatric assent varied considerably (between 5 and 13 years) even within North America. Furthermore, only 14% of studies specifying a minimal age actually provided a justification for their choice [16, 29, 41, 42]. This variability is in keeping with the broader literature in which some authors propose a “school-age threshold”, suggesting “considerable capacities of 5–7 year-old children” [43] while others advocate that children less than 11 years of age have a limited understanding of research information [34]. This inconsistency in reported ages for obtaining assent adds further complexity to the numerous challenges already encountered regarding standardization of ethical procedures in international research collaborations [44]. Inconsistent assent descriptions and procedures add to the existing variability in research ethics boards requirements and cultural contexts for informed consent and may contribute to selection bias of included patients and further limit generalizability of results in such collaborations [45, 46].

Although age is an admittedly important factor in determining a child’s ability to provide assent, it is also important to consider other factors such as cognitive ability when assessing a child’s potential for understanding medical information. However, our review found that the vast majority of studies enrolling children (92.4%) did not specify criteria other than age for obtaining assent and over 90% of articles did not report on cognitive abilities. Interestingly, European assent guidelines published in 2016 detailed an approach to the assent process in children including a lower age limit but did not address either capacity or competence [47]. This is important given that a child’s cognitive abilities may affect both their competence and capacity to provide assent for research. Competence is a legal definition that refers to the mental ability of an individual to make medical decisions and is usually determined by a judge. Capacity, on the other hand, is a functional assessment made by a clinician regarding an individual’s ability to make an informed decision in a particular situation [10, 48]. It is important to note that although determination of competence and capacity involve different procedures, the results of both assessments directly impact the rights of a child to provide input into research participation. For example, if the child is deemed not competent and/or incapable of providing assent, then the rights of the child are effectively overridden. The United Nations Convention on the Rights of the Child allows for this provision in Article 12 by stating “the views of the child being given due weight in accordance with the age and maturity of the child.”

The distinction between capacity and competence is of particular importance for inclusion of specialized populations in research, such as children with intellectual disabilities, developmental delays or critical illness. Children with severe development delay are unlikely to be competent or have capacity to provide assent under most circumstances. Interestingly, the Gillick Competence (based on United Kingdom case law), states that the process of obtaining assent depends not only on the maturity and intelligence of the child but is also conditional on the seriousness of the situation [7]. For example, a previously healthy 12 year-old with severe septic shock may be deemed competent to provide assent but would not have the capacity to do so while critically ill as demonstrated by O’Hearn et al. who showed that very few patients were considered approachable for assent (i.e. they lacked capacity) during the first 24 h of their PICU admission [49]. Similarly, a six-year-old may have the capacity to provide assent for a study on the use of band aids versus sutures for small cuts but would likely not be able to provide assent for a study of life-saving therapies such as being put on a heart-lung machine.

Another important finding from studies operationalizing assent is that the assent procedure itself was rarely detailed with the majority of studies (83.5%) not specifying what information was provided to the participants. This raises questions about how often meaningful engagement of the child in the assent process actually occurred. This is especially important in view of the fact that studies have reported that a child may not wish to participate in a specific study despite their parent’s consent [22, 37] therefore highlighting the importance of obtaining and documenting assent obtained from pre-adolescent children. Interestingly, 85% and 99% of studies did not refer to specific ethical or legal guidelines respectively, despite existing recommendations from professional societies [3, 4] and legal bodies [5, 7, 11].

A limitation of this scoping review is that the search strategy only identified articles using “assent” in their title or abstract. This pragmatic approach identified approximately half of the articles with the word “assent” anywhere in the article. However, a random assessment of 100 articles with the word “assent” in the full-text but not the abstract or title found only one article providing further details on the assent process. Therefore, it is likely that our screening process included the majority of articles describing the assent process. Another possible limitation of our review is that the sparsely reported assent rates, documentation of approaches to assent and references to guiding documents may have reflected the limited word-count allowed for most publications and/or lack of journal requirements for reporting details on assent. However, it is interesting that despite literature over the years emphasizing the importance of reporting assent in publications [50], the International Committee of Medical Journal Editors recommendations on the Protection of Research Participants does not specifically require reporting on assent [51]. Finally, as none of the studies reporting assent discussed the effect of the requirement or result of the assent process on their study enrollment or results.

## Conclusion

We acknowledge that individual children, settings and jurisdictions may require different approaches to obtaining assent. However, there should be agreement on important principles to follow for obtaining assent with resulting guidance on assessing capacity, delivering information and documenting the assent process. Future steps might include organizing jurisdiction specific focus groups to obtain input from all relevant stakeholders (e.g. research ethics boards, researchers, lawyers, parents, children, physicians and allied health personnel) in order to obtain agreement surrounding the minimal process needed to obtain meaningful assent.

## Supplementary information

**Additional file 1**: References for articles analyzed for Scoping Review.

## Data Availability

All data generated and/or analyzed during the current study are available in this published article (and its additional files) and the referenced articles.
