# Supplementary material for: Operationalization of assent for research participation in pre-adolescent children: a scoping review
Source: BMC Med Ethics. 2022 Nov 3;23:106. doi: 10.1186/s12910-022-00844-2 (PMC9632024; doi:10.1186/s12910-022-00844-2)
Supplement: Supplementary file 1 — Additional file 1: References for articles analyzed for Scoping Review. [file 12910_2022_844_MOESM1_ESM.docx]

**Additional references for included citations (alphabetical order).**

1. Adcock KG, Hogan SM, Elci OU, Mills KL. Do Illustrations Improve Children's Comprehension of Assent Documents? J Pediatr Pharmacol Ther. 2012;17: 228-35.
2. Ali S, Rajagopal M, Klassen T, Richer L, McCabe C, Willan A et al; KidsCAN PERC Innovative Pediatric Clinical Trials No OUCH Study Team. Study protocol for two complementary trials of non-steroidal or opioid analgesia use for children aged 6 to 17 years with musculoskeletal injuries (the No OUCH study). BMJ Open. 2020;10: e035177.
3. Barned C, Dobson J, Stintzi A, Mack D, O'Doherty KC. Children's perspectives on the benefits and burdens of research participation. AJOB Empir Bioeth. 2018;9: 19-28.
4. Baseke J, Musenero M, Mayanja-Kizza H. Prevalence of hepatitis B and C and relationship to liver damage in HIV infected patients attending Joint Clinical Research Centre Clinic (JCRC), Kampala, Uganda. Afr Health Sci. 2015;15: 322-7.
5. Berry DC, Neal M, Hall EG, McMurray RG, Schwartz TA, Skelly AH et al. Recruitment and retention strategies for a community-based weight management study for multi-ethnic elementary school children and their parents. Public Health Nurs. 2013;30: 80-6.
6. Botha MH, van der Merwe FH, Snyman LC, Dreyer G. The vaccine and cervical cancer screen (VACCS) project: acceptance of human papillomavirus vaccination in a school-based programme in two provinces of South Africa. S Afr Med J. 2015;105: 40-3.
7. Brink Y, Cockcroft J, Seedat S, May P, Kalberg W, Louw Q. The postural stability of children with foetal alcohol spectrum disorders during one-leg stance: A feasibility study. Afr J Disabil. 2018;7: 319.
8. Broadhurst MJ, Kelly JD, Miller A, Semper A, Bailey D, Groppelli E et al. ReEBOV Antigen Rapid Test kit for point-of-care and laboratory-based testing for Ebola virus disease: a field validation study. Lancet. 2015;386: 867-74.
9. Broome ME, Richards DJ. The influence of relationships on children's and adolescents' participation in research. Nurs Res. 2003;52: 191-7.
10. Brown LR, Barber S, Benson PE, Littlewood S, Gilthorpe MS, Wu J et al. PLATOON: Premature Loss of bAby Teeth and its impact On Orthodontic Need - protocol. J Orthod. 2019;46: 118-125.
11. Browne S, Kechadi MT, O'Donnell S, Dow M, Tully L, Doyle G et al. Mobile Health Apps in Pediatric Obesity Treatment: Process Outcomes From a Feasibility Study of a Multicomponent Intervention. JMIR Mhealth Uhealth. 2020;8: e16925.
12. Burns SK, Hendriks J, Mayberry L, Duncan S, Lobo R, Pelliccione L. Evaluation of the implementation of a relationship and sexuality education project in Western Australian schools: protocol of a multiple, embedded case study. BMJ Open. 2019;9: e026657
13. Chandrakantan A, Reinsel RA, Jasiewicz R, Jacob ZC, Seidman PA. An exploratory study of the relationship between postoperative nausea and vomiting and post discharge nausea and vomiting in children undergoing ambulatory surgery. Paediatr Anaesth. 2019;29: 353-360.
14. Chatzipantazi P, Roy KM, Cameron SO, Goldberg D, Welbury R, Bagg J. The feasibility and acceptability of collecting oral fluid from healthy children for anti-HCV testing. Arch Dis Child. 2004;89: 185-7.
15. Coussens M, Destoop B, De Baets S, Desoete A, Oostra A, Vanderstraeten G, Van Waelvelde H, Van de Velde D. A Qualitative Photo Elicitation Research Study to elicit the perception of young children with Developmental Disabilities such as ADHD and/or DCD and/or ASD on their participation. PLoS One. 2020;15: e0229538.
16. Mahesh KM, John D, Rose A, Paul P. Prevalence of ocular morbidity amongtribal children in Jawadhi hills, southern India: A cross-sectional study. Indian J Ophthalmol. 2019;67: 386-390.
17. Malaty HM, Abudayyeh S, O'Malley KJ, Wilsey MJ, Fraley K, Gilger MA, Hollier D, Graham DY, Rabeneck L. Development of a multidimensional measure for recurrent abdominal pain in children: population-based studies in three settings. Pediatrics. 2005;115: e210-5.
18. Mangochi H, Gooding K, Bennett A, Parker M, Desmond N, Bull S. How should assent to research be sought in low income settings? Perspectives from parents and children in Southern Malawi. BMC Med Ethics. 2019;20: 32.
19. Mbhatsani VH, Mbhenyane XG, Mabapa SN. Development and Implementation of Nutrition Education on Dietary Diversification for Primary School Children. Ecol Food Nutr. 2017;56: 449-461.
20. Mengoni SE, Irvine K, Thakur D, Barton G, Dautenhahn K, Guldberg K, Robins B, Wellsted D, Sharma S. Feasibility study of a randomised controlled trial to investigate the effectiveness of using a humanoid robot to improve the social skills of children with autism spectrum disorder (Kaspar RCT): a study protocol. BMJ Open. 2017;7: e017376.
21. Miller VA, Baker JN, Leek AC, Drotar D, Kodish E. Patient involvement in informed consent for pediatric phase I cancer research. J Pediatr Hematol Oncol. 2014;36: 635-40.
22. Miner JR, Kletti C, Herold M, Hubbard D, Biros MH. Randomized clinical trial of nebulized fentanyl citrate versus i.v. fentanyl citrate in children presenting to the emergency department with acute pain. Acad Emerg Med. 2007;14: 895-8.
23. Mori K, Torii H, Fujimoto S, Jiang X, Ikeda SI, Yotsukura E, Koh S, Kurihara T, Nishida K, Tsubota K. The Effect of Dietary Supplementation of Crocetin for Myopia Control in Children: A Randomized Clinical Trial. J Clin Med. 2019;8: 1179.
24. Muzaffar H, Raffaelli M, Teran-Garcia M, Wiley A, Gonzalez M, Hannon BA. A Community-Based Participatory Assessment of the Health Status and Obesity Risks in Children From Rural Farmworker Families in the Midwest. Hisp Health Care Int. 2019;17: 149-155.
25. Nunes LM, Ribeiro R, Niewiadonski VDT, Sabino E, Yamamoto GL, Bertola DR et al. A new insight into CFTR allele frequency in Brazil through next generation sequencing. Pediatr Pulmonol. 2017;52: 1300-1305.
26. Ondrusek N, Abramovitch R, Pencharz P, Koren G. Empirical examination of the ability of children to consent to clinical research. J Med Ethics. 1998;24: 158-65.
27. Piloya T, Bakeera-Kitaka S, Kekitiinwa A, Kamya MR. Lipodystrophy among HIV-infected children and adolescents on highly active antiretroviral therapy in Uganda: a cross sectional study. J Int AIDS Soc. 2012;15: 17427.
28. Rehman H, Naveed S, Usmanghani K. Efficacy and safety of Linkus, Aminophylline diphenhydramine and acefyllin piperazine for the treatment of cough in children. Pak J Pharm Sci. 2016;29: 1027-32.
29. Rossi GN, Sorazabal AL, Salgado PA, Squassi AF, Klemonskis GL. Toothbrushing procedure in schoolchildren with no previous formal instruction: variables associated to dental biofilm removal. Acta Odontol Latinoam. 2016 Apr;29(1):82-89. English.

# Shah RR, Patil PH. Effect of Gross Motor and Fine Motor Exercises on Trunk Control in Subjects with Spastic Cerebral Palsy. Indian Journal of Forensic Medicine and Toxicology. 2020;14: 608-613.

1. Susman EJ, Dorn LD, Fletcher JC. Participation in biomedical research: the consent process as viewed by children, adolescents, young adults, and physicians. J Pediatr. 1992;121: 547-52.
2. Sweeney T, Hegarty F, Powell K, Deasy L, Regan MO, Sell D. Randomized controlled trial comparing Parent Led Therapist Supervised Articulation Therapy (PLAT) with routine intervention for children with speech disorders associated with cleft palate. Int J Lang Commun Disord. 2020;55: 639-660.
3. Syms CA 3rd, Grantham ML. Otologic iontophoresis: a no-papoose technique. Ann Otol Rhinol Laryngol. 2013;122: 487-91.
4. Tait AR, Voepel-Lewis T, Malviya S. Do they understand? (part II): assent of children participating in clinical anesthesia and surgery research. Anesthesiology. 2003;98: 609-14.
5. Taljaard DS, Leishman NF, Eikelboom RH. Personal listening devices and the prevention of noise induced hearing loss in children: the Cheers for Ears Pilot Program. Noise Health. 2013;15: 261-8.
6. Theiss-Nyland K, Qadri F, Colin-Jones R, Zaman K, Khanam F, Liu X, Voysey M, Khan A, Hasan N, Ashher F, Farooq YG, Pollard AJ, Clemens JD. Assessing the Impact of a Vi-polysaccharide Conjugate Vaccine in Preventing Typhoid Infection Among Bangladeshi Children: A Protocol for a Phase IIIb Trial. Clin Infect Dis. 2019;68: S74-S82.
7. Traube DE, Cederbaum JA, Kerkorian D, Bhupali C, McKay MM. African American children's perceptions of HIV-focused community-based participatory research. J Empir Res Hum Res Ethics. 2013;8: 79-90.
8. Treble-Barna A, Patronick J, Uchani S, Marousis NC, Zigler CK, Fink EL, Kochanek PM, Conley YP, Yeates KO. Epigenetic Effects on Pediatric Traumatic Brain Injury Recovery (EETR): An Observational, Prospective, Longitudinal Concurrent Cohort Study Protocol. Front Neurol. 2020;11: 460.
9. Trout AT, Dillman JR, Xanthakos S, Kohli R, Sprague G, Serai S, Mahley AD, Podberesky DJ. Prospective Assessment of Correlation between US Acoustic Radiation Force Impulse and MR Elastography in a Pediatric Population: Dispersion of US Shear-Wave Speed Measurement Matters. Radiology. 2016;281: 544-552.
10. Unguru Y, Sill AM, Kamani N. The experiences of children enrolled in pediatric oncology research: implications for assent. Pediatrics. 2010;125: e876-83.
11. Vanderby SA, Babyn PS, Carter MW, Jewell SM, McKeever PD. Effect of anesthesia and sedation on pediatric MR imaging patient flow. Radiology. 2010;256: 229-37.
12. Varma S, Jenkins T, Wendler D. How do children and parents make decisions about pediatric clinical research? J Pediatr Hematol Oncol. 2008;30: 823-8.
13. von Baeyer CL, Chambers CT, Forsyth SJ, Eisen S, Parker JA. Developmental data supporting simplification of self-report pain scales for preschool-age children. J Pain. 2013;14: 1116-21.
14. Wolthers OD. A questionnaire on factors influencing children's assent and dissent to non-therapeutic research. J Med Ethics. 2006;32: 292-7.
15. Zacharczuk GA, Toscano MA, López GE, Ortolani AM. Evaluation of 3Mix-MP and pulpectomies in non-vital primary molars. Acta Odontol Latinoam. 2019;32: 22-28. English.
